# Raw Meat-Based Diets in Dogs and Cats

**DOI:** 10.3390/vetsci4030033

**Published:** 2017-06-28

**Authors:** Maria Fredriksson-Ahomaa, Tiina Heikkilä, Noora Pernu, Sara Kovanen, Anna Hielm-Björkman, Rauni Kivistö

**Affiliations:** 1 Department of Food Hygiene and Environmental Health, Faculty of Veterinary Medicine, P.O.Box 66, 00014 University of Helsinki, Finland; tiina.e.heikkila@helsinki.fi (T.H.); noora.pernu@helsinki.fi (N.P.); sara.kovanen@helsinki.fi (S.K.); rauni.kivisto@helsinki.fi (R.K.); 2 Department of Equine and Small Animal Medicine, Faculty of Veterinary Medicine, P.O.Box 66, 00014 University of Helsinki, Finland; anna.hielm-bjorkman@helsinki.fi

**Keywords:** raw food, zoonotic enteric pathogens, PCR, dogs, cats

## Abstract

Feeding pets raw meat-based diets (RMBDs) is commonly practiced by many companion animal owners and has received increasing attention in recent years. It may be beneficial for the animals, but may also pose a health risk for both pets and their owners, as RMBDs may be contaminated by enteric pathogens—such as *Campylobacter*, *Salmonella,* and *Yersinia*—which are the most common zoonotic bacteria causing enteritis in humans. Little information exists on the prevalence of these pathogens in pet food, and thus one aim was to investigate the prevalence of *Campylobacter*, *Salmonella*, and *Yersinia* in commercial RMBDs from retail stores. Little evidence also exists on the significance of raw meat feeding on the shedding of *Campylobacter*, *Salmonella*, and enteropathogenic *Yersinia* in the feces of pets, and therefore, the second goal was to study the presence of these pathogens in dogs and cats fed RMBDs. Polymerase chain reaction (PCR) only sporadically detected *Campylobacter, Salmonella*, and enteropathogenic *Yersinia* in RMBDs. These pathogens were not found by culturing, indicating a low contamination level in frozen RMBDs. They were also detected in the feces of dogs and cats, but the association with feeding RMBDs to them remained unclear.

## 1. Introduction

Most dogs and cats are fed commercially produced dry (pellets/kibble) or wet (canned) food, but the popularity of raw-meat based diets (RMBDs) is rising [1,2]. Sometimes RMBDs are called BARF (Bones And Raw Food or Biologically Appropriate Raw Food) or RAP (Raw Animal Products) [3,4]. RMBDs can contain skeletal muscle, fat, internal organs, cartilage, and bones from farm animals (ruminants, pigs, and poultry), horses, game, and fish. RMBDs can be prepared at home or sold commercially. The most common form of commercial RMBDs are frozen, but freeze-dried products have recently also entered the market [3,5]. Some are intended to be nutritionally complete and balanced, but products intended only for supplemental feeding also exist [3].

Proponents of feeding RMBDs to their pets claim important health benefits for the diets, such as improvement of coat and skin, and reduction in dental diseases [3]. However, health risks, such as enteric pathogens in raw meat, an unbalanced diet and internal punctures caused by bones in the food, may also occur [3]. Raw meat and internal organs can easily be contaminated already during slaughter through feces and tonsils, which are frequently positive for *Campylobacter, Salmonella*, and *Yersinia *[6,7]. Raw poultry is most commonly contaminated by *Campylobacter* and *Salmonella*, and raw pork by *Yersinia* and *Salmonella* [6,8]. Therefore, pet food containing raw meat may also be contaminated with these bacterial pathogens. Still, only a limited number of studies are available on the prevalence of these enteric pathogens in RMBDs (Table 1). 

Campylobacteriosis, salmonellosis, and yersiniosis are the most commonly reported notifiable gastroenteritis in the European Union (EU) [27]. The diseases are typically foodborne, especially through eating or handling undercooked or raw meat products. Contact with pets is also considered a risk factor for infection. These pathogens are widespread in nature and found in the gastrointestinal tract of wild and domestic mammals and birds. They have also been reported in dogs and cats with and without diarrhea [14,16,28]. However, the reported prevalence rates vary highly between the studies (Table 1). *Campylobacter* is the most commonly found zoonotic pathogen in the feces of both dogs and cats. They mainly carry *C. upsaliensis*, which very rarely causes campylobacteriosis in humans. *C. helveticus*, which is typically not associated with human disease, has frequently been isolated from cats (Table 1). 

The goals of our study were to investigate the prevalence of *Campylobacter*, *Salmonella*, and enteropathogenic *Yersinia* in commercial RMBDs and to determine whether dogs and cats given daily RMBDs excrete these enteric bacteria in their feces. 

## 2. Materials and Methods

### 2.1. Samples and Sample Preparation

In total, 88 RMBDs originating from 12 producers were bought from retail shops in 2015 and 2016 and studied before expiration dates. All producers were from Finland and only domestic meat was used in the products, which were intended for dogs and/or cats. The most common raw meat in commercial RMBDs were beef (43%) and poultry (41%), followed by pork (27%) (Table 2). Twenty-one RMBDs contained more than one raw meat source. In addition to skeletal muscle, some of the diets included organs and bone/cartilage. All RMBDs were frozen and thawed at room temperature before screening with polymerase chain reaction (PCR) and culturing. 

A total of 50 fecal samples from 29 dogs fed raw meat and 21 dogs not fed raw meat during 2013 and 2014. All fecal samples from dogs were stored at −70 °C until PCR screening and culturing. The dogs fed raw meat received only commercial or home-made RMBDs daily and the dogs not fed raw meat received only dry pellets daily several months before sampling. The dogs did not have signs of diarrhea.

A total of 75 fecal samples were collected from two indoor cats living in the same household, 47 samples of which were obtained from the older cat (born 19 April 2015) between 3 November 2015 and 28 November 2016 and 28 from the younger cat (born 22 January 2016) between 13 May 2016 and 28 November 2016. Both cats have received RMBDs daily and have lived indoor their whole lives. During the fecal sampling time from 3 November 2015 to 28 November 2016, 48 samples from 38 RMBDs fed to the cats were studied (Table 2). Poultry (61%) was the most common meat type in the diets, followed by pork (32%) and beef (29%).

Approximately a 10-g food sample was removed from the packaging and mixed thoroughly with the 90 mL of buffered peptone water (BPW, LAB M, Kerava, Finland) in the stomacher bag by hand or by a stomacher blender (LabBlender 400, London, UK) for 1 min. About a 1-g fecal sample was mixed with 9 mL BPW shortly with a vortex. All samples were incubated at 37 °C overnight (16–18 h) before DNA extraction.

### 2.2. PCR Screening of Pathogens

The presence of *Campylobacter, Salmonella*, and enteropathogenic *Yersinia* was screened by real-time PCR based on SYBRGreen [29]. DNA was isolated from 1 mL of the ON enrichment by ZR Fecal DNA MiniPred™ (Nordic BioSite Oy, Helsinki, Finland) according to the manufacturer’s instructions. Two µL of the DNA was added to 18 µL of mastermix (iQ^TM^ SYBRGreen Supermix, BioRad) containing primers specific for *Campylobacter* [30], *Salmonella* [31], and enteropathogenic *Yersinia* [32,33]. The threshold cycle (Ct) under 39 and a specific melting temperature indicated a positive result. Additionally, *ail*-positive samples were run separately with *ail* primers for *Y. enterocolitica* [32] and *Y. pseudotuberculosis *[33]. *Campylobacter* (*rrn*)-positive samples were separately studied with primers specific for *C. jejuni* (*map*) and *C. coli* (*ceu*) [34]. 

### 2.3. Isolation of Campylobacter, Salmonella, and Yersinia

The presence of *Salmonella *and* Yersinia *were studied by enrichment and selective agar plates [29]. For *Salmonella* isolation enrichment on semisolid Rappaport-Vassiliadis (MSRV, Labema) for 24 h at 42 °C was used before inoculating on selective xylose-lysine-deoxycholate (XLD, Labema) plates which were incubated for 24 h at 37 °C. Cold enrichment at 6 °C for at least three weeks was used for *Yersinia* isolation. After cold enrichment, 10 µL was inoculated on CIN plates which were incubated at 30 °C for 20–24 h. Up to four typical colonies on XLD and CIN plates were sub-cultured on blood agar plates and identified with API 20E (BioMerieux, France). Serotyping was done with commercial antisera (Denka Seikan, Japan). The presence of *Campylobacter* was studied by plating (1 g feces/1 mL 0.9% saline) on modified charcoal cefoperazone deoxycholate agar (mCCDA) with cefoperazone and amphotericin (Oxoid, Basingstoke, UK) and identification was done by gram staining and PCR according to Olkkola et al. [28].

### 2.4. Statistical Analyses

Statistical analyses were performed using the analytical software package SPSS^®^ Statistics Version 24 (IBM Corp., Armonk, NY, USA). Fisher’s exact test was used to analyze the relation between feeding RMBDs and dry pellets and the presence of *Campylobacter*,* Salmonella*, and enteropathogenic *Yersinia*.

## 3. Results

Enteric pathogens were detected in 28% of the RMBDs, originating from 12 producers (Table 3). *Campylobacter* was the most frequent pathogen, detected by PCR in 15% of the RMBDs. However, all samples were negative for *C. jejuni* and *C. coli*. *Y. enterocolitica* carrying the *ail* gene was detected in 11% of the samples. *Salmonella* was detected in only 2% of the samples and, surprisingly, *Y. pseudotuberculosis* was also detected in two samples.

*Campylobacter* was most frequently found in RMBDs containing beef (21%) and *Y. enterocolitica* in RMBDs containing pork (15%) (Table 4). The two *Y. pseudotuberculosis*-positive samples were both from raw poultry products, one of which was also *Salmonella* positive. All samples were culture negative.

*Campylobacter* was detected by PCR in 55% of the tested fecal samples from 29 dogs fed raw meat and in 33% fecal samples from 21 dogs fed dry pellets (Table 5). *Campylobacter* was clearly, but not significantly (*p* = 0.158), more frequently detected in dogs fed RMBDs than in dogs fed dry pellets. Only a weak positive association (Phi = 0.216) was observed between dogs fed raw meat and the presence of *Campylobacter*. Of the dogs fed raw meat, two (7%) shed *Salmonella* and one (3%) shed *ail*-positive *Y. enterocolitica*. 

*Campylobacter* was isolated from 20 dogs and most (90%) of them shed *C. upsaliensis*. *C. jejuni *was isolated from two dogs, and *Salmonella* and *ail*-positive *Y. enterocolitica* from one dog each. All these three pathogens were isolated only from dogs fed RMBDs. 

Both cats were sampled between 10 May 2016 and 28 November 2016, and *Campylobacter* was detected in all (100%; 26/26) fecal samples from the old cat and in most (96%; 27/28) samples from the young cat by PCR and *C. helveticus* was isolated from both cats. Between 3 November 2015 and 3 May 2016, before the young cat came to the household, the old cat was *Campylobacter*-positive only once (5%; 1/21). 

*Y. enterocolitica* was detected in two fecal samples from the old cat in January, and *Y. enterocolitica* of bioserotype 4/O:3 was isolated from both samples. In June 2016, during the same sampling day, *Salmonella* was detected from both cats by PCR, but not by culturing.

## 4. Discussion

Pets are important members of many households, and several health benefits from pet ownership have been documented, including reduction in loneliness and depression. Despite the benefits, companion animals may be one potential source of zoonotic infections to the owners. Feeding RMBDs, a current trend among dog and cat owners, may transmit zoonotic meat-borne pathogenic bacteria to pets via contaminated RMBDs. However, only a few studies have been conducted on the prevalence of zoonotic enteropathogenic bacteria, such as *Salmonella*, *Campylobacter*, and *Yersinia*, in RMBDs (Table 1). In our study, *Campylobacter* was detected by PCR in 15% of the commercial RMBDs. However, no *C. jejuni* or *C. coli*, which are typical human pathogenic species, were identified, showing that these pathogens appear to be rare in pet food, at least in Finland where the prevalence of *Campylobacter* is below 10% in broilers at retail level (https://www.evira.fi/en/about-evira/news/2016/campylobacter-most-prevalent-in-poultry-in-late-summer/). *Y. enterocolitica* carrying the *ail*-gene was detected in 11% of the samples and more often in RMBDs containing pork. Fattening pigs at slaughter carry pathogenic *Y. enterocolitica* frequently in their tonsils, and raw pork is considered the main infection source [35]. *Salmonella* was rarely detected in our study and the occurrence (2% by PCR and <1% by culturing) was clearly lower than reported in Canada and USA (Table 1). One reason is probably the very low (<1%) prevalence of *Salmonella* in Finnish farm animals, including poultry [36]. All samples in our study were negative by culturing, indicating that the number of zoonotic enteric bacteria is low in domestic frozen RMBDs. The bacteria may also be unculturable due to prolonged stress during storage in the freezer.

Pets fed raw meat are suspected to shed meat-borne pathogens more often in their feces than pets not fed raw meat. In our study, dogs fed raw meat shed *Campylobacter* more frequently than dogs fed dry pellets; however, the difference was not statistically significant. Furthermore, the most common species isolated from dog feces was *C. upsaliensis*, and *C. jejuni* was rarely isolated. *Campylobacter* is a very common finding, and *C. upsaliensis* is the most common species found in dog feces also in several other studies (Table 1). Dogs and cats are shown to be the main reservoirs for *C. upsaliensis* [12,14,18,20]. *C. upsaliensis* appears to be commensal in dogs and cats, and no differences between the isolation rates in healthy and diarrhoeic animals have been reported [12,17]. However, their lifestyle may affect the shedding of pathogens in the feces. Dogs exposed to outdoor water sources have an increased risk of shedding *C. upsaliensis *[10]. In our study, the association between feeding raw meat and shedding *C. upsaliensis* remained unclear; however, *C. jejuni* was only isolated from dogs fed RMBDs. *Salmonella* and *Y. enterocolitica* were rarely detected in the dog feces in our study. Both pathogens were isolated from only one dog each; however, both positive dogs were fed RMBDs. In other studies, the isolation rates of *Salmonella* in dog feces varied between 1% and 23% (Table 1). Lentz et al. demonstrated that dogs fed raw meat were more likely to shed *Salmonella* in their feces than dogs not fed raw meat [25]. Joffe and Schlesinger demonstrated that dogs fed raw chicken shed *Salmonella* in their feces, and they also showed that raw chicken was frequently contaminated with *Salmonella* [37]. 

We also studied two indoor cats fed RMBDs on a daily basis the whole life, and were able to demonstrate that ever since the young cat (three months old) came to the household, both cats shed *Campylobacter* in the feces. All samples from the old cat and all but one from the young cat were *Campylobacter*-positive from 5 October 2016 until 28 November 2016, when we stopped the sampling. *C. helveticus* was isolated from the feces of both cats, which is the species mostly found in cats [12,14]. It is possible that the young cat brought the *Campylobacter* to the household and infected the older one. Younger animals (under one year) are more likely to shed *Campylobacter*, as they may be more susceptible to colonization than older individuals [20]. However, the old cat shedding *Campylobacter* in its feces for over five months until the end of the sampling was also an interesting observation in our study. In an earlier study, long-term colonization of *Campylobacter* spp. has been described in dogs and cats [38]. In our study, during the period that the cats shed *Campylobacter* in the feces,* Campylobacter* was also detected more frequently in the food fed to the cats; however, the link between eating raw meat and shedding *Campylobacter* remained unclear.

*Salmonella* and enteropathogenic *Yersinia* were rarely detected in the cat feces and RMBDs fed to them in our study. However, *Y. enterocolitica* belonging to bioserotype 4/O:3, which is the most common human pathogenic type typically transmitted by raw or undercooked pork, was surprisingly isolated twice from the old cat’s feces before the young cat came to the household. The cat has only lived indoors and was fed RMBDs containing pork, so most possibly the transmission to the cat occurred through contaminated food. Raw pork is a potential source of *Y. enterocolitica* 4/O:3 infections in dogs and cats [39]. *Salmonella* was detected in both cats on the same sampling time, indicating that RMBDs fed to them could be the infection source.

This study shows that RMBDs can contain zoonotic pathogens that can be a health hazard both to pets and in-contact humans; however, a clear link between feeding RMBDs and infections in pets and pet owners still remains mostly unclear [5,24,26,37,40]. As feeding RMBDs gains popularity, the potential risks should be reviewed, and lists of precautions and typical disease symptoms should be made available to dog owners feeding their pets RMBDs. However, consumers should handle all dog food products carefully, being mindful of their potential risks to human and animal health. Several outbreaks of human *Salmonella* infections have been caused by contaminated dry dog foods [41,42,43]. One reason that dry-food outbreaks have been reported more often than RMBDs outbreaks might be because feeding RMBDs is still marginal compared to feeding dry food. A study of in-household simultaneous canine and human fecal pathogen testing combined with clinical symptoms is now under way (Hielm-Björkman, personal communication).

## 5. Conclusions

Zoonotic meat-borne bacteria—such as *Campylobacter, Salmonella*, and enteropathogenic *Yersinia*—were only sporadically detected in RMBDs by PCR. These pathogens were not found by culturing, indicating a low contamination level in frozen commercial RMBDs produced in Finland. *C. upsaliensis* was a common finding in dogs irrespective of feeding RMBDs or dry pelletst. *Salmonella* and enteropathogenic *Yersinia* were detected only in dogs fed RMBDs; however, the infection source and transmission routes remained unclear. *Y. enterocolitica* bioserotype 4/O:3 and *Salmonella* were probably transmitted from contaminated RMBDs to the indoor cats but not *C. helveticus*. However, the indoor cats shed *C. helveticus* in their feces for months. The practice of feeding raw meat to dogs and cats may increase the potential transmission risk of meat-borne pathogens to people. Pet owners, especially individuals at increased risk for infectious diseases (small children, old people, and immunocompromised individuals), should be aware of the safety risks of feeding RMBDs. Attention should particularly be paid to storage and handling of raw meat. RMBDs should be kept frozen until use.

## Figures and Tables

**Table 1:** The prevalence of *Campylobacter, Salmonella*, and *Yersinia enterocolitica* in feces or rectal swabs of dogs and cats and in commercial raw meat-based diets (RMBDs).

Sample	No.	Country	*Campylobacter*				*Salmonella*	*Yersinia Enterocolitica *^a^	Reference
			spp.	*Jejuni*	*Upsaliensis*	*Helveticus*			
Dogs	138	Canada					23%		[9]
	251	Canada	43%	6%	37%		1%		[10]
	1212	China						8%	[11]
	190	Finland	28%	9%	17%				[12]
	4325	Germany ^**b**^						4%	[13]
	147	Ireland	43%	10%	29%				[14]
	171	Italy	17%	9%	5%				[15]
	90	New Zealand	36%	13%	23%	1%			[16]
	529	Norway	23%	3%	20%				[17]
	290	Spain	35%	14%	21%				[18]
	180	Sweden	37%	4%	29%	1%			[19]
	249	UK	38%	1%	38%				[20]
	126	UK					1%		[21]
	130	USA	1%				2%		[22]
	554	USA					5%		[23]
Cats	84	Finland	32%	8%	7%	17%			[12]
	2624	Germany ^c^						0.3%	[13]
	35	Ireland	42%	9%	26%	6%			[14]
	102	Italy	15%	8%	6%	1%			[15]
	110	New Zealand	16%	5%	5%	7%			[16]
	301	Norway	18%	3%	13%				[17]
RMBDs	25	Canada	<4%				20%		[24]
	40	Canada	<3%				5%		[25]
	50	New Zealand	28%	22%					[16]
	196	USA					8%		[5]
	240	USA	<0.4%				7%		[26]

**^a^** pathogenic bioserotype or *ail* positive; **^b^** mainly Germany (77%), and 13 other European countries (33%); **^c^** mainly Germany (81%), and 10 other European countries (19%).

**Table 2:** Raw meat-based diets (RMBDs) from different producers studied in 2015 and 2016.

Producer	No. of RMBDs	Fed to		Number of Samples Containing						
		Dogs	Cats	Poultry	Pork	Beef	Sheep	Horse	Game	Fish
A	6	6	0	1	3	2	0	0	1	1
B	43	26	17	22	8	13	1	2	1	6
C	10	8	2	1	1	9	0	0	0	0
D	1	1	0	0	1	0	0	0	0	0
E	9	6	3	0	4	8	0	0	0	1
F	8	2	6	7	4	3	0	0	0	0
G	1	1	0	0	0	1	0	0	0	1
H	5	0	5	3	0	2	0	0	0	0
I	1	0	1	0	1	0	0	0	0	0
J	1	0	1	1	0	0	0	0	0	0
K	2	0	2	0	2	0	0	0	0	0
L	1	0	1	1	0	0	0	0	0	0
All	88	50	38	36	24	38	1	2	2	9

**Table 3:** Occurrence of zoonotic enteric bacteria in raw meat-based diets.

Producer	Number of Samples	Number of PCR-Positive Samples				
		All	*Campylobacter*	*Salmonella*	*Yersinia Enterocolitica*	*Yersinia Pseudotuberculosis*
A	6	1	0	0	1	0
B	43	14	8	1	6	0
C	10	0	0	0	0	0
D	1	0	0	0	0	0
E	9	2	2	0	0	0
F	8	2	0	0	1	1
G	1	1	0	0	1	0
H	5	2	1	0	1	0
I	1	0	0	0	0	0
J	1	1	1	0	0	0
K	2	1	1	0	0	0
L	1^a^	1	0	1	0	1
All	88	25	13	2	10	2

^a^ One sample containing poultry from producer L was contaminated with *Salmonella* and *Y. pseudotuberculosis*.

**Table 4:** Occurrence of zoonotic enteric bacteria in different meats used in raw meat-based diets.

Meat	Number of Samples	Number of PCR-Positive Samples				
		All	*Campylobacter*	*Salmonella*	*Yersinia Enterocolitica*	*Yersinia Pseudotuberculosis*
Beef	38	9	8	0	1	0
Pork	24	8	4	0	4	0
Poultry	36	8	4	1	4	2
Sheep	1	0	0	0	0	0
Horse	2	1	0	1	1	0
Game	2	0	0	0	0	0
Fish	9	5	3	0	2	0

**Table 5:** Detection of zoonotic enteric bacteria in fecal samples of dogs fed raw meat-based diets (RMBDs) or dry pellets.

Feeding	Number of Dogs	Number of Dogs Shedding					
		*Campylobacter*		*Salmonella*		*Y. Enterocolitica*	
RMBDs	29	16	(55%)	2	(7%)	1	(3%)
Dry pellet	21	7	(33%)	0		0	
